# Measurement, Education and Tracking in Integrated Care (METRIC): use of a culturally adapted education tool versus standard education to increase engagement in depression treatment among Hispanic patients: study protocol for a randomized control trial

**DOI:** 10.1186/s13063-017-2109-y

**Published:** 2017-08-03

**Authors:** Katherine Sanchez, Brittany H. Eghaneyan, Michael O. Killian, Leopoldo Cabassa, Madhukar H. Trivedi

**Affiliations:** 1 School of Social Work, University of Texas at Arlington, 211 South Cooper Street, Arlington, TX 76019 USA; 2 George Warren Brown School of Social Work, Washington University in St. Louis, Campus Box 1196, One Brookings Drive, St. Louis, MO 63130-4899 USA; 3 Department of Psychiatry, UT Southwestern Medical Center, 5323 Harry Hines Blvd., Dallas, TX 75390-9119 USA

**Keywords:** Depression, Education, Hispanics, Stigma, Primary care, iPad screening, *Fotonovela*, Measurement-based integrated care, Care management

## Abstract

**Background:**

Significant mental health disparities exist for Hispanic populations, especially with regard to depression treatment. Stigma and poor communication between patients and their providers result in low use of antidepressant medications and early treatment withdrawal. Cultural factors which influence treatment decisions among Hispanics include fears about the addictive and harmful properties of antidepressants, worries about taking too many pills, and the stigma attached to taking medications. Primary care settings often are the gateway to identifying undiagnosed or untreated mental health disorders, particularly for people with co-morbid physical health conditions. Hispanics, in particular, are more likely to receive mental healthcare in primary care settings. Recent recommendations from the U.S. Preventive Services Task Force are that primary care providers screen adult patients for depression only if systems are in place to ensure adequate treatment and follow-up.

**Methods:**

We are conducting a randomized controlled trial among 150 depressed adult Hispanics in a primary care safety net setting, testing the effectiveness of a culturally appropriate depression education intervention to reduce stigma and increase uptake in depression treatment among Hispanics, and implement a Measurement-Based Integrated Care (MBIC) model with collaborative, multidisciplinary treatment and culturally tailored care management strategies.

**Discussion:**

This study protocol represents the first randomized control trial of the culturally adapted depression education *fotonovela*, *Secret Feelings*, among Hispanics in a primary care setting. The education intervention will be implemented after diagnosis using an innovative screening technology and enrolled in measurement-based integrated care for the treatment of depression, which will help build the evidence around cultural adaptations in treatment to reduce mental health disparities.

**Trial registration:**

ClinicalTrials.gov, NCT02702596. Registered on 20 March 2016.

**Electronic supplementary material:**

The online version of this article (doi:10.1186/s13063-017-2109-y) contains supplementary material, which is available to authorized users.

## Background

### Chronic disease and depression

Chronic medical conditions affect nearly half of the people in the United States and two-thirds of encounters with health professionals are for the management of those conditions [1]. Effective treatment of chronic illness is complicated and requires significant investment on the part of the patients and their families. More than 300 million people worldwide were living with depression in 2015, making it the leading cause of medical disability in the world, causing significant disease burden and medical cost [2]. One in six Americans is afflicted in their lifetime and chronicity is common [3].

Adults with chronic medical conditions have high rates of depression and anxiety which often impair self-care and compliance with treatment of their disease [4]. For patients with diabetes, depression severity is associated with diabetes complications, medical co-morbidity, greater anxiety, dysthymia, financial worries, social stress, and poorer quality of life [5]. Major depression increases the burden of chronic illness by increasing perception of symptoms, causing additional impairment in functioning, and increasing medical cost through over-utilization of the healthcare system [4]. Many patients with co-morbid depression and chronic disease may sabotage their own treatment by “focusing on the physical” and delaying treatment of mental health concerns for weeks or months. Such delays in addressing the underlying depression cannot only make remission difficult, but can make treatment of the physical condition challenging [6].

### Risk factors for Hispanics and depression

A national sample of Hispanics has estimated the prevalence of depression among the population to be 27% [7]. Hispanics have double the risk of co-morbid depression and diabetes, with rates as high as 33%, compared with the general population [8]. Response to depression treatment in Hispanics is slow and relapse rates are high, which may explain the early discontinuation of medication without consulting their provider [9, 10]. Hispanic patients often voice fears about the addictive and harmful properties of antidepressants, worries about taking too many pills, and the stigma attached to taking medications [11, 12]. In the context of co-morbid chronic disease, depression can be difficult to detect by primary care providers, especially where screening for depression is inconsistent, creating further barriers to treatment [13].

Lack of English fluency is associated with reduced healthcare use [14]. Limited English proficiency (LEP), limited health literacy, geographic inaccessibility, and lack of medical insurance are all more common among immigrants, minority populations, individuals of low socioeconomic status, and people in rural areas. The citizenship, or legal status of an individual, impacts the extent to which they can secure quality healthcare, as does the level of acculturation and duration of residence in the U.S. [15]. Individuals with LEP are less likely to self-identify a need for mental health services, which subsequently predicts lifetime mental healthcare use, and results in longer duration of untreated disorders [16, 17].

### Integrated care

Integrated care is a systematic approach to the treatment of depression in primary care that involves systematic screening for depression, the integration of a care manager, with primary care provider oversight and psychiatric consultation, as needed, to more proactively treat mental health problems [4]. The primary function of the care manager is to manage the mental health disorders of the patients, monitor their response to treatment, and facilitate communication between the patient, the primary care providers, and the consulting psychiatrist [18, 19]. The functions of the care manager can address significant barriers to treatment such as stigma, limited knowledge of mental health disorders, poor doctor–patient communication, and treatment engagement and adherence, which disproportionately affect racial and ethnic minorities [19, 20]. However, those functions may be limited unless culture specific strategies are employed to engage and retain minority patients and those with LEP in treatment [21, 22].

### Patient education

While patient education has been established as essential in chronic disease management [23], less is known about its role in engagement and improved outcomes for mental disorders [24]. Low health literacy is related to poor management of chronic diseases, lack of basic knowledge about medical conditions and treatments, less understanding and use of preventive services, worse health outcomes, and higher rates of hospitalization and emergency care use [25]. However, qualitative evaluation of conceptual models which include comprehensive educational interventions for depressed Hispanics have found patients were optimistic about the benefits of treatment [26], especially those which adapt materials for literacy and cultural content, and include homework materials that are linguistically and idiomatically appropriate [5].

### Aims and objectives

The study aims to examine a novel culturally adapted patient education intervention to increase engagement by Hispanics in depression treatment. The intervention will be implemented after diagnosis using an innovative screening technology and via measurement-based integrated care for the treatment of depression, which will have a substantive impact on reducing mental health disparities in the Hispanic population.

#### Specific aim 1

Examine the specific effects of a Depression Education *Fotonovela* (DEF) to increase knowledge of depression, reduce stigma, and increase engagement in depression treatment among Hispanic patients.

#### Specific aim 2

Evaluate the impact of measurement-based integrated care (MBIC) on retention in treatment and remission of depression symptoms in one community-based Federally Qualified Health Center (FQHC) in North Texas whose patient population is majority Hispanic.

#### Specific aim 3

Examine the feasibility of universal screening and accurate detection of depression among adult primary care patients utilizing innovative iPad Depression Screening technology.

## Methods/Design

### Setting and participants

The study will take place at one FQHC whose mission is to provide a community medical home through accessible, compassionate, and quality healthcare services. The center offers a full range of comprehensive primary and preventive services, including childhood immunizations and physical exams, adult physical exams, management of chronic illnesses, family planning services, maternity care services, and health education promotion. In 2015, the center served 11,895 unduplicated patients for a total of 25,362 patient visits. Of those patients, 90% were Hispanic and the majority did not speak English. There are currently five physicians and four mid-level providers (physician assistants and nurse practitioners) working at the center.

The target enrollment is 150 patients over the 24-month enrollment period. All adult primary care patients at the center will be asked to participate in screening for depression with the iPad Depression Screening application (universal screening). After being informed about the expectations for participation in the study and the compensation payments, it is expected that a minimum of 2–3 patients per week will agree and be enrolled in the study. Patients who meet study inclusion criteria will be enrolled on a first-come, first-serve basis until the target of 150 patients is reached. The study protocol was reviewed and approved by the Institutional Review Board of the University of Texas at Arlington. All patients will provide written informed consent to participate.

### Intervention

**Fig. 1:**
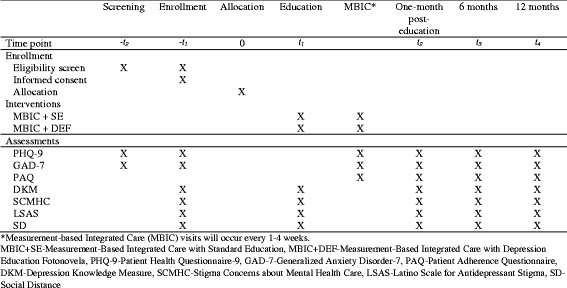
Schedule of enrollment, interventions and assessments

**Fig. 2:**
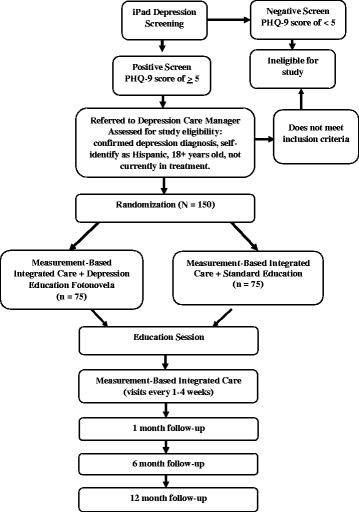
Study flow diagram

The study will compare outcomes among patients randomly assigned to one of two treatment conditions: (1) MBIC with DEF (MBIC + DEF); or (2) MBIC with standard education (MBIC + SE). All patients enrolled in the Measurement, Education and Tracking in Integrated Care (METRIC) study will receive MBIC. The schedule of enrollment, interventions, and assessments is outlined in Fig. 1, study flow is outlined in Fig. 2, and page listings for all study components can be found in the Standard Protocol Items: Recommendations for Interventional Trials (SPIRIT) Checklist (see Additional file 1).

#### Depression Education Fotonovela

**Fig. 3:**
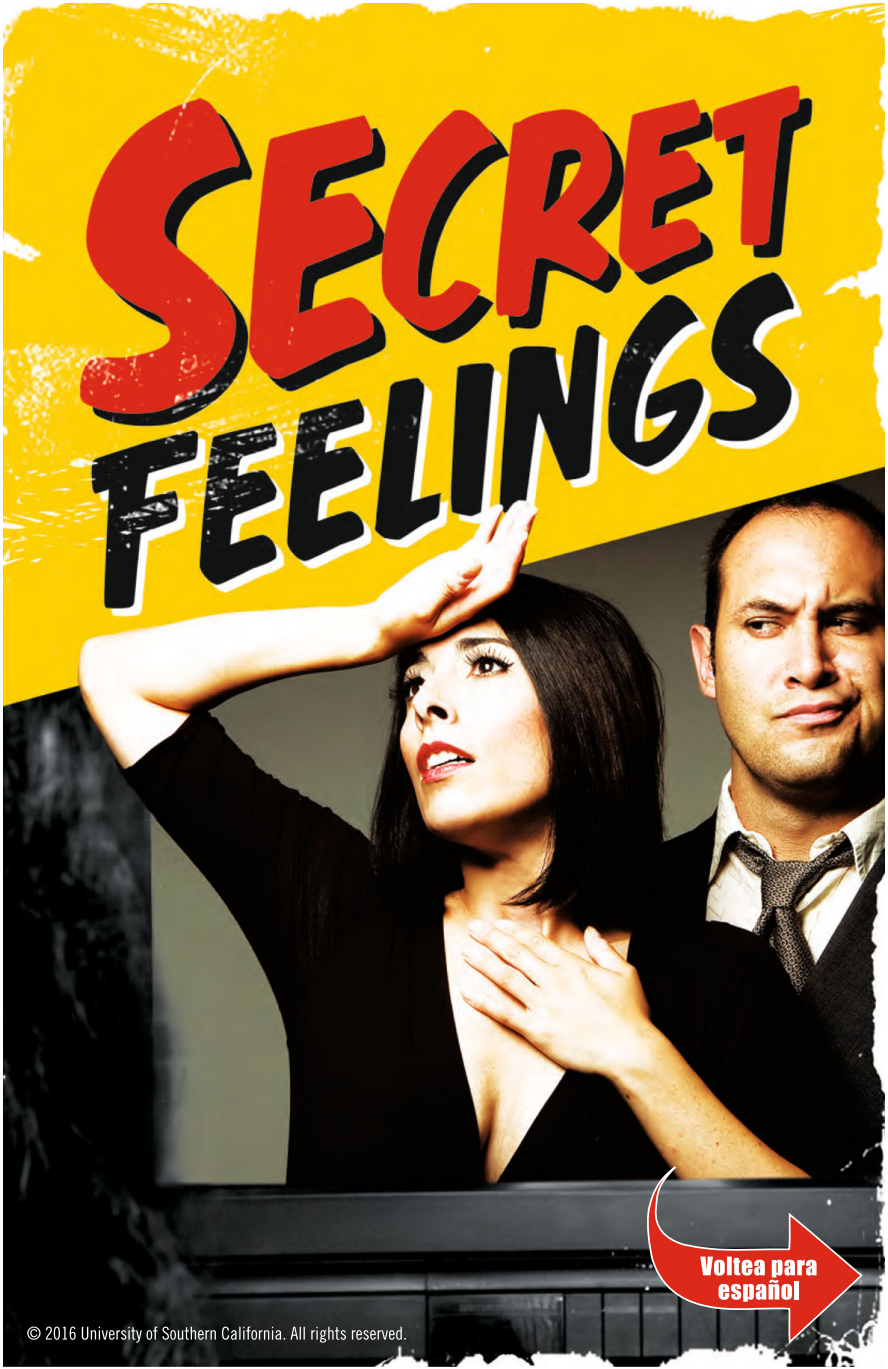
Depression *fotonovela* cover–English

**Fig. 4:**
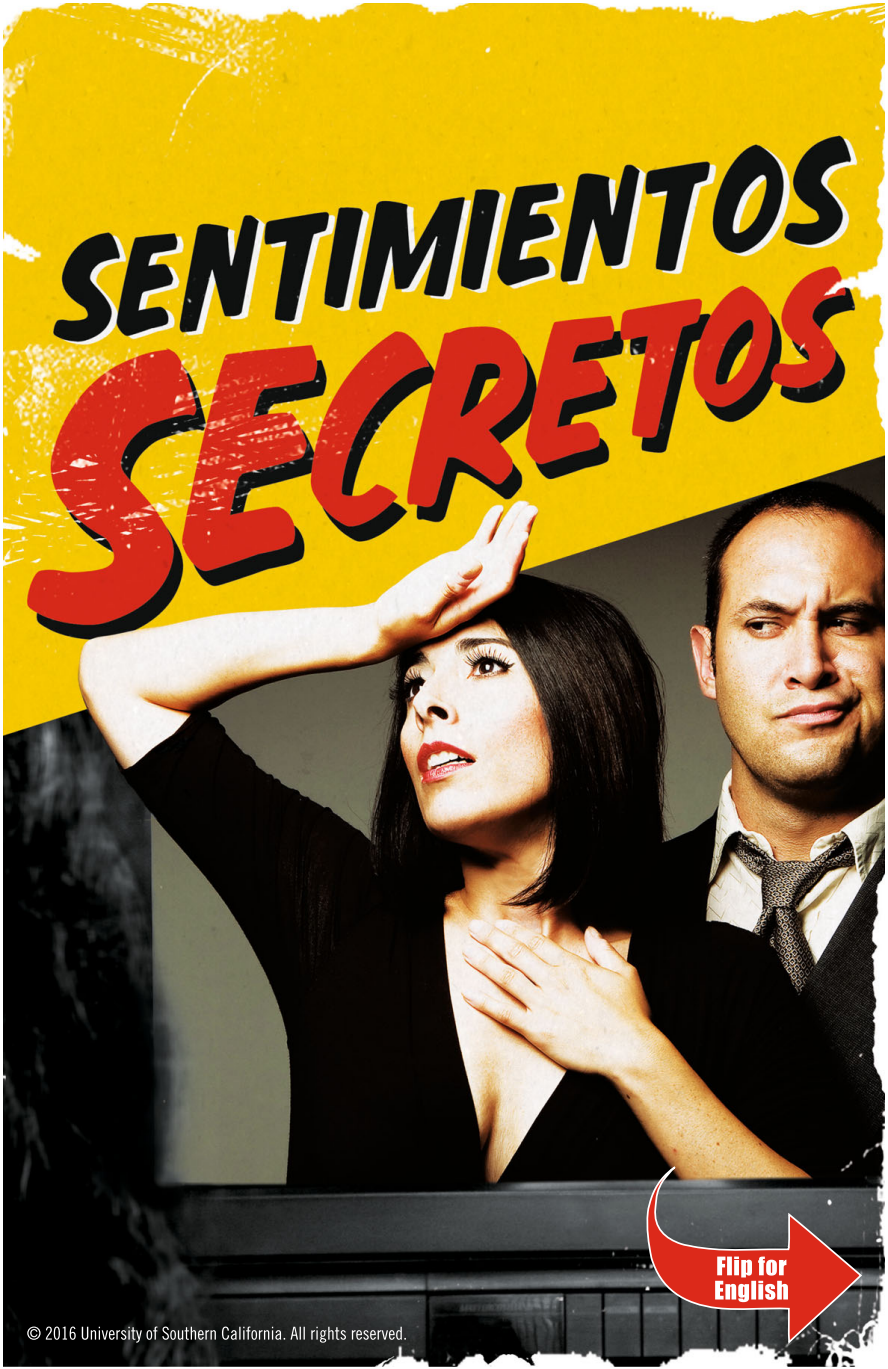
Depression *fotonovela* cover–Spanish

This study will test a unique, culturally adapted DEF titled *Secret Feelings*, developed by Cabassa, Molina, and Baron [27]. Developed as a comic-book style pamphlet using photographs and dialogue bubbles, the *fotonovela* is written at a 4th-grade reading level and presents information on depression symptoms and treatment while portraying a soap opera-style dramatic story. This *fotonovela* is an Entertainment-Education (EE) approach which has demonstrated early success as an effective tool for engaging Hispanic audiences and increasing knowledge about specific health issues [28–30]. The DEF differs from typical patient education materials by incorporating surface and deep-level cultural elements including the use of simple language, attractive visuals, cultural norms, and educational messages that target specific misconceptions and attitudes about depression and depression treatment common among Hispanics [30, 31]. See Figs. 3 and 4 for sample images of the *fotonovela* covers.

The DEF is designed to enhance the awareness and understanding of depression, its role in chronic disease, its impact on Hispanic populations, and the multitude of barriers to effective treatment in a culturally meaningful way. The *fotonovela* has demonstrated significant improvements in depression knowledge and reductions in stigma toward antidepressants and mental health treatment in a community education setting and a multiservice community clinic setting but has not been tested as a depression treatment engagement in a primary care setting [32, 33].

#### Measurement-based integrated care

This project will implement MBIC, which synthesizes the science of integrated healthcare and incorporates the strategies of measurement-based care, often missing from existing models [34]. The MBIC intervention is based on the principles of collaborative care developed by Unutzer and Katon [35] at the University of Washington and adapted to include measurement-based treatment strategies developed by Trivedi and colleagues [36]. Abundant evidence supports the conclusion that a measurement-based approach significantly improves outcomes and lowers costs, especially when coupled with care managers. Although Trivedi et al. [36] have led successful development of measurement-based strategies, measurement-based care is still seldom used in treating patients with depressive disorders. The concept of MBIC for the treatment of depression includes: the systematic assessment of symptoms; antidepressant treatment side effects and medication adherence; and the incorporation of a depression care manager (DCM) to track patients and keep them engaged in care using behavioral interventions.

In this study, a DCM will engage the patient in treatment, assess barriers to treatment, and develop solutions to barriers. The DCM will be a bilingual, licensed Masters level clinical social worker (LCSW) and have specialized training in brief psychotherapy and interventions demonstrated effective in primary care settings, such as Problem Solving Treatment for Primary Care (PST-PC) [37] and Behavioral Activation (BA) [38]. The DCM will also work closely with the patients’ primary care provider (PCP) in the development and execution of treatment plans which will include counseling and/or pharmacotherapy. Using measurement-based care treatment principles, patients’ depressive symptoms will be measured at defined intervals, along with antidepressant treatment side effects and treatment adherence (if applicable). Measuring mental health symptoms at the start of a patient’s treatment and regularly thereafter facilitates treatment planning and adjustment, indicates when it is time to start or change medications, avoids patients staying on ineffective treatments for too long, and assists PCPs in knowing when to refer for consultation or get additional help.

### Study procedures

#### Universal screening with iPad technology

The project will utilize a novel technology to systematically identify depressed patients, quantify the severity of their depression, and guide treatment recommendations. The web-based software platform is designed to support the use of MBIC by providing feedback to providers at the point of care. All adult primary care patients will be asked to complete the iPad Depression Screening (universal screening) at the annual visit and at new/non-acute visits. During the routine intake process, designated clinical staff will give the patient the iPad and ask them to complete the screening for depression. The iPad Depression Screening will be presented in English or Spanish, depending on the patient’s preference. The first screen in the iPad application is presented to record consent and the patient will be informed that refusal to consent for screening will have no effect on the care they receive at the clinic. If the patient does not consent, the screening process will be terminated and the patient will continue with their scheduled visit.

The iPad Depression Screening application begins with the nine-item Patient Health Questionnaire (PHQ-9) [39]. If the patient screens positive for depression, indicated by a score of ≥ 5, the screening application immediately offers the Generalized Anxiety Disorder (GAD-7) [40] anxiety measure. Once completed, an electronic notification is generated to the DCM and patient’s PCP and the application screen will lock with a message to return the iPad to a clinic staff member.

Immediately after a patient screens positive for depression, the medical assistant will document the PHQ-9 score in the patient’s electronic health record (EHR) and will inform the provider that the patient screened positive for depression to enable the provider to interpret the depression score. Patients who screen positive for depression but previously had a diagnosis of depression and are in treatment will be excluded from enrollment in the study. Because of the specificity of the stigma measurements and the *fotonovela* being tested, self-identified non-Hispanic patients will also be excluded from enrollment in the study. Figure 2 summarizes the screening, study eligibility, randomization, group allocation, and study flow.

#### Randomization

After a patient screens positive for depression with the iPad Depression Screening application, the provider will immediately facilitate an introduction to the DCM for potential enrollment in the study. The DCM will assess for the presence of the nine diagnostic criteria for major depression from the Diagnostic and Statistical Manual of Mental Disorders (DSM-IV) [41] through a clinical interview. Once the depression diagnosis is confirmed, patients will be offered the opportunity to participate in the study, guided through the informed consent process and randomized to one of two treatment groups. A block randomization list that randomizes 150 participants into 75 blocks will be generated through an online randomization website and will be kept in a locked filing cabinet in the DCM’s office. Patients will be assigned to the group that coordinates with their patient enrollment number on the randomization list. One hundred and fifty patients will be randomly assigned to two possible treatment arms: (1) MBIC + DEF; or (2) MBIC + SE. A randomization log will be maintained by the Project Coordinator, which will include treatment assignment, names of participants, and participant identification numbers.

At the baseline visit, the DCM will have the patient complete all study measures and engage the patient during the regular intake process. The DCM will document the patient’s PHQ-9 screening score, the stigma measures, current treatment preferences (pharmacotherapy, counseling), and potential barriers to care in a research database. The DCM will also explain the compensation schedule, which includes payment for completing measures after the education intervention and each follow-up research visit, and schedule the patient’s education visit and first MBIC appointment to take place after the education visit.

#### Education

Within one week, patients will return to the clinic to complete their education session and study measures with a research assistant – either SE or DEF, depending on the group to which the patient was randomized. Two bilingual research assistants, hired from the school of social work, will be assigned and trained to deliver the different education interventions (one each, without exposure to the other education materials). Patients will be encouraged to bring family members or loved ones to the education session. Each education session will be provided in English or Spanish, depending on patient preference, and will conclude with compensation to the patient upon completion of the study measures.

##### Standard education

The research assistant trained in SE will read the patient education materials from the National Institute of Mental Health (NIMH) [42] which includes: What is depression? What causes depression? Signs and symptoms. Who is at risk? Treating and living with depression. The SE materials emphasize the complexity of depression that involves thoughts and feelings, behaviors, and physical symptoms, and issues of co-morbid illness and depression as a biological disease. The SE education materials are printed in English and Spanish. The SE session will last about 20 min.

##### Depression education fotonovela

The research assistant trained in the DEF will read *Secret Feelings* (*Sentimientos Secretos*) with the patient, including the question and answer page at the back of the booklet. The story is presented in both English and Spanish at a 4th-grade reading level. After reading the *fotonovela* together, the patients will be asked if they have any additional questions. The DEF session will last for about 30–45 min.

#### Measurement-based integrated care

As part of MBIC, patients will meet with the DCM every 1–4 weeks, depending on their treatment plans, to complete brief psychotherapy interventions. Mental health measures and medication response and adherence will be collected using the iPad Depression Screening and Monitoring application and will be recorded in the EHR.

##### Follow-up visits

Patients will return to the clinic to complete all study measures and be compensated at the following time points: one-month post education session, six months post enrollment, and 12 months post enrollment. Between follow-up visits, research personnel will contact patients monthly to remind them of study participation and future appointments. If patients do not return for their follow-up visits, data will be collected from their medical record to include PHQ-9 scores and medication information, if available.

##### Intervention fidelity and data monitoring

Each research team member will undergo depression education training with the Principal Investigator and protocol and data collection training with the Project Coordinator. Research assistants will meet with the Principal Investigator and perform a final, practice education session prior to depression education intervention visits with participants. Additionally, education sessions will be audio recorded (with patients’ consent) and regularly reviewed by the Principal Investigator to assure adherence to the protocol. The Project Coordinator will perform data audits quarterly to assure data collection accuracy.

### Measures/Assessments

Demographic information will be collected from each participant to include gender, age, marital status, level of education, and co-morbid medical conditions (self-report). Three brief mental health measures will be collected via the iPad Depression Screening and Monitoring application technology by clinical staff and transferred to the research database by research staff. All research and clinical trial data will be collected via REDCap (Research Electronic Data Capture), a secure, web-based application used for a variety of research and maintained by Vanderbilt University Office of Research [43]. All measures have been validated in Spanish.

#### Patient Health Questionnaire-9 (PHQ-9)

The PHQ-9 is a self-report measure that assesses the frequency of symptoms on each of the nine DSM-IV [41] criteria for depression for “the last two weeks.” Responses for each item are scored as 0 for “not at all,” 1 for “several days,” 2 for “more than half the days,” and 3 for “nearly every day,” which results in a range of possible scores of 0–27. Total scores of 5–9 represent mild depression, 10–14 represent moderate depression, 15–19 represent moderately severe depression, and > 20 represent severe depression. Patients with a PHQ-9 score ≥ 10 are considered to have clinically significant depressive symptoms. The PHQ-9 has been shown to be a reliable and valid measure of depression severity in primary care samples with a Cronbach’s alpha of 0.89 and has demonstrated construct validity among African American, Hispanic, and non-Hispanic white populations [44, 45]. The PHQ-9 is designed to provide necessary information about potential depression diagnoses while not overburdening patients and providers. The PHQ-9 will be administered via the iPad screening and monitoring application at screening and all follow-up MBIC and research visits.

#### Generalized Anxiety Disorder-7 (GAD-7)

The GAD-7 is a seven-item self-report scale for identifying the presence of generalized anxiety disorder (GAD) and assessing symptom severity. The items of the GAD-7 are based on the diagnostic criteria for GAD in the DSM-IV and assess frequency of symptoms over the last two weeks using four response options: “not at all,” “several days,” “more than half the days,” and “nearly every day,” scored as 0, 1, 2, and 3, respectively. A score of 10 or greater on the GAD-7 represents a point for identifying cases of GAD. Cut points of 5, 10, and 15 represent mild, moderate, and severe levels of anxiety, respectively. The reliability of the GAD-7 in primary care settings, as well as in the general population, has demonstrated excellent internal consistency reliability with a Cronbach’s alpha of 0.92 and 0.89, respectively [40]. The GAD-7 will be administered after a positive screen for depression and at all follow-up visits. The GAD-7 will be administered via the iPad screening and monitoring application at screening at all follow-up MBIC and research visits.

#### Patient Adherence Questionnaire (PAQ)

The PAQ [46] is a two-item measure of adherence to antidepressant medication over the past week, possible changes in dose made by the patient without consultation with their provider, and reason for the change (sample response: “I reduced my dose because I felt better”). The PAQ is scored as 0 for one day or less of missing the medication, and 1 for days > 1 missed, and each reason endorsed for changes made in dose. Any score greater than ≥ 1 is considered “non-adherent to treatment” and will prompt the provider to address adherence. The PAQ will be administered via the iPad screening and monitoring application at all follow up MBIC and research visits for those patients who are prescribed medication.

#### Depression Knowledge Measure (DKM)

The DKM [32] is a 17-item measure that evaluates symptom recognition and treatment knowledge. Symptom recognition is assessed with a list of ten depression symptoms (sleeping too little, eating too much, feeling agitated, feeling guilty, and loss of interest) and five non-depressive symptoms (hearing voices, being full of energy, being violent, having hallucinations, and feeling confident). Scoring for this section allocates 1 point for each symptom that is correctly identified as a depression symptom or not a depression symptom. Treatment knowledge is measured with seven true–false questions from the Depression Literacy Questionnaire (D-Lit) measure adapted for use with Hispanic populations [13, 47]. The D-Lit has demonstrated internal consistency and test–retest reliability [47, 48] and has been used with immigrant populations [49]. Each correctly answered item is given a score of 1 point. With ten symptom recognition items and seven treatment knowledge items, the DKM scores range from 0 (all incorrect) to 17 (all correct). The measure will be administered at baseline, after the education session, at the one-month post-education session, and at the six-month and 12-month research follow-up visits.

Three brief stigma measures will be used to measure key stigma constructs that deter depression treatment utilization among the Hispanic population. These measures have demonstrated validity and reliability among Spanish-speaking, Hispanic primary care patients [50]. The three scales will be administered at baseline, after the education session, one-month post education session, and at the six-month and 12-month research follow-up visits.

#### Stigma Concerns about Mental Health Care (SCMHC)

The SCMHC [50] is a three-item measure that assesses stigma-related barriers to depression treatment utilization (sample item: “I would not want to receive treatment for depression because of being embarrassed to talk about personal matters with others”). Adapted from a larger measure of barriers to depression treatment [51, 52], response options are scored as 0 = “Disagree,” 1 = “Agree,” and 7 = “don’t know/refuse.” Scores for the measure are calculated by taking the sum of the three items (except for the items answered “don’t know/refuse”), with total score in the range of 0–3. The criterion-related and predictive validity of the three-item measure has been supported with a sample of Latinos diagnosed with depression and has demonstrated good internal consistency reliability with a Cronbach’s alpha of 0.84 [50].

#### Latino Scale for Antidepressant Stigma (LSAS)

The LSAS [50] is a seven-item measure designed to assess stigma specifically related to the use of antidepressant medications among Latinos. Respondents are asked to state how most people think with regard to stigma-related statements pertaining to use of antidepressants (sample item: “Prescription medicines for depression are for people who are not strong”). Possible response items are scored 0 = “No one thinks that way,” 1 = “Some people think that way,” 2 = “Everyone thinks that way,” and 7 = “Don’t know or refuse to answer.” The LSAS score is calculated by taking the sum of the seven items (except for the items answered as “don’t know or refuse to answer,” with total scores in the range of 0–14. LSAS scores have been correlated with depression treatment utilization and the scale has demonstrated good internal consistency reliability with a Cronbach’s alpha of 0.80 [50].

#### Social Distance (SD) scale

The SD is a six-item scale, which measures social distance desirability from someone with depression or history of depression treatment (sample item: “Would you be friends with someone who is or had been in treatment for depression?”) [53, 54]. Social distance is a widely measured stigma construct studied in mental health research [55]. Possible response items are scored 0 = “no,” 1 = “maybe,” 2 = “yes,” and 7 = “don’t know/refused.” Scores are calculated by taking the sum of items 1–6 (except for items answered with “don’t know/refuse”), with total scores in the range of 0–12. Lower scores indicate greater social distance. The relationship between SD scores and depression treatment engagement has been previously reported in a sample of Latinos with depression [50]. Previous studies have reported excellent internal consistency reliability with caregivers of patients with bipolar disorders [56]. The SD has demonstrated adequate internal consistency reliability with a Cronbach’s alpha of 0.75.

### Data analysis

#### Sample size

A total of 150 participants will be randomly assigned to the treatment and control groups. Intervention and control groups, estimated at 56 participants each, are required to have an 80% chance of detecting an effect size of 0.57 (power = 0.80) with a two-sided 5% significance level between the two group on depression scores. The effect size of 0.57 was calculated in a similar multisite randomized controlled trial of depression among women (N = 205) in an OB-GYN clinic with a similarly racially diverse, mixed socioeconomic status population [57]. Assuming an attrition rate of 25%, 70 participants for each group would be needed in the current study, which represents a cautious estimation. A systematic review of interventions to increase recovery from depression in primary care settings found attrition rates at the 12-month follow-up visit to be in the range of 6–18% [58].

#### Analysis

In addition to the study measures, additional data collected from the EHR will include demographics (age, marital status, race/ethnicity, insurance status) and medical co-morbidity. Planned data analyses include bivariate analyses of baseline data to compare demographic and clinical characteristics of the two intervention groups. In order to examine the success of randomization, t-tests and chi-square tests will be used to check for between-group equivalence. For each dependent variable, intent-to-treat longitudinal analyses will be conducted and outcomes for the two intervention groups will be compared. Mixed-design analysis of variance (repeated measures ANOVA with a between-subjects factor) tests will assess for within-group changes over time and for significant differences between the MBIC + DEF and MBIC + SE groups for each of the various outcome measures. Between-group differences in the likelihood in engagement in depression treatment (depression treatment and other treatment modalities) will be tested through chi-square tests followed by binary logistics regression modeling. Engagement in treatment will be dichotomously coded. All tests will be two-tailed with *p* < 0.05 used to determine significance. Study aims 1 and 2 will be tested for the MBIC + DEF versus MBIC + SE. All models will be checked for the need for additional covariates (demographic and clinical baseline characteristics) and interaction terms. Analyses will use all available data (i.e. intent-to-treat analyses) from participants with baseline data, post education (DEF or SE) visits and 12-month follow-up visits.

The evaluation of Aim 1 will examine the specific effects of the MBIC + DEF to increase knowledge about depression, reduce stigma, and increase engagement in depression treatment compared to MBIC + SE as measured by the following:

1. Changes in knowledge of depression among Hispanic patients pre and post DEF compared with SE, as measured by the DKM. The DEF group is expected to report significantly greater DKM scores over time when compared with the SE group indicating gains in depression knowledge following receipt of depression education.
2. Changes in attitudes among Hispanic patients toward depression pre and post DEF compared with SE using the SCMHC, LSAS, and the SD measures. The DEF group is expected to report significant changes in SCMHC, LSAS, and the SD over time when compared to the SE group and reported reductions in stigma related to mental health and psychiatric medications following depression education.
3. Determine engagement in depression treatment for patients in DEF vs. SE.
  - Quantify the number of patients diagnosed with depression and received the DEF intervention who went on to begin depression treatment compared with SE. The DEF group is hypothesized to have a greater portion of individuals engaging in depression treatment than the SE group following treatment. Furthermore, participants in the DEF group will have an increased likelihood of later engaging in treatment following the intervention.
  - Quantify which treatment modality the patient with depression chose after the DEF compared to SE: pharmacotherapy; counseling; or other behavioral intervention. The DEF group is hypothesized to have a greater portion of individuals engaging in pharmacotherapy, counseling, or other behavioral interventions than the SE group following treatment. Furthermore, participants in the DEF group will have an increased likelihood of later engaging in pharmacotherapy, counseling, or other behavioral interventions following the depression.

The primary outcome for Aim 2 is the change from baseline to 12-month scores on the PHQ-9 depression measure (dependent variable) for patients enrolled in MBIC. Outcomes will then be compared between and the MBIC + DEF and the MBIC + SE intervention groups.

1. Current literature on the PHQ-9 suggests that a 5-point drop in mean depression scores is an indication of a clinically significant response to treatment [59]. The DEF group is hypothesized to have a greater proportion of the group report a clinically significant decrease in depression scores (5-point decrease in PHQ-9 scores) when compared with the SE group.
2. The U.S. Department of Health and Human Services suggests that the objective of collaborative care research should be to have 40% or more of the patients realize a 50% or greater reduction in depression scores [60].

The evaluation of Aim 3 will examine the feasibility of universal screening for depression using the iPad Depression Screening and Monitoring technology, and will be measured by its positive predictive value, clinical utility, ease of use, and acceptability of routine use among clinicians:

1. Quantify the number of adult patients screened per month and if the score is being placed in the EHR.
2. Determine if the iPad Depression Screening application changes PCP approach to the diagnosis and treatment of depression as measured by the number of patients with a new depression diagnosis as a percentage of the total number of adult patients seen in the clinic compared to a matched set the year prior to the study based on EHR (baseline data).
3. Quantify changes in the number of patients with a depression diagnosis being treated with antidepressants by the PCP as a percentage of the total number of patients seen in the clinic compared to a matched set the year prior to the study based on EHR (baseline data).

## Discussion

This study protocol describes a randomized control trial testing a culturally adapted depression education intervention designed to reduce stigma and increase disease literacy. The target of the three-part design is to improve detection, diagnosis, and increasing uptake in depression treatment among Hispanics in an integrated care model in a community-based health center. This study addresses the need for both identification and elimination of the barriers to treatment for the most common mental health condition affecting Hispanics. Various other design options were discussed, including testing the *fotonovela* as a discrete intervention, without the context of integrated care. However, with the U.S. Preventive Services Task Force recommendation for universal screening for depression in primary care settings and the emphasis on systems to address positive screens [61], the rationale for testing an innovative technology to facilitate screening and implementing an evidence-based treatment model for which there is a lack of studies among minority populations offers a greater opportunity to translate established research into clinical practice. The rationale for designing a complete intervention, while still testing a specific element for which cultural values about mental health may be distinct, appears scientifically feasible, and shows scientific promise.

The strengths and weakness of using a SE control group were carefully considered prior to selection. The primary benefit of the SE control group is that it ensures a comparable contact across the two interventions. As a result, a significant effect observed in the *fotonovela* condition cannot be attributed to differences in contact or participation outcome expectations. A potential concern with the control group is a threat to internal validity due to lack of treatment fidelity and interventionist allegiance. To reduce these concerns, the SE intervention will be manualized (based on NIMH education materials) and intervention fidelity procedures will be the same across the two interventions.

The scope of this project is ambitious, in that we aim to change provider behavior and screen all adult primary care patients for depression. With that in mind, we realize that we will identify a large number of patients whose depression was previously unrecognized. For busy primary care practitioners, this will undoubtedly increase their workload. However, with the support of the DCM and MBIC, the burden of systematic measurement, education about the disease and tracking of outcomes will be reduced. Additionally, we look to offset the potential treatment burden by training the PCPs and their clinic staff in evidence-based treatment guidelines, and through the use of the iPad Depression Screening and Monitoring application. It is anticipated that the recognition and treatment of depression will improve other co-morbid chronic disease and ultimately reduce the overall burden of disease management on the primary care practice.

### Trial status

Enrollment for METRIC commenced in February 2016. The estimated completion date of enrollment is January 2018, with 12-month follow-up and data lock by January 2019.

## Additional file

Additional file 1: SPIRIT Checklist: Recommended items to address in a clinical trial protocol and related documents*. (DOC 122 kb)

## Abbreviations

BA: Behavioral activation
DCM: Depression care manager
DEF: Depression education *fotonovela*
DKM: Depression Knowledge Measure
EHR: Electronic health record
FQHC: Federally qualified health center
GAD-7: Generalized Anxiety Disorder-7
HHS: U.S. Department of Health and Human Services
LCSW: Licensed clinical social worker
LEP: Limited English proficiency
LSAS: Latino Scale for Antidepressant Stigma
MBIC + DEF: Measurement-based integrated care with depression education *fotonovela*
MBIC + SE: Measurement-based integrated care with standard education
MBIC: Measurement-based integrated care
METRIC: Measurement, Education, and Tracking in Integrated Care
NIMH: National Institute of Mental Health
PAQ: Patient Adherence Questionnaire
PCP: Primary care provider
PHQ-9: 9-item Patient Health Questionnaire
PI: Principal Investigator
PST-PC: Problem Solving Treatment for Primary Care
SCMHC: Stigma concerns about mental health care
SD: Social Distance Scale
SE: Standard education
